# Atomistic Insights on Interactions Between Sulfur-Containing Pollutants and PMMA: A Semiempirical, DFT, SAPT and Molecular Dynamics Study

**DOI:** 10.3390/polym18101199

**Published:** 2026-05-14

**Authors:** Dušica Krunić, Stevan Armaković, Maria M. Savanović, Sanja J. Armaković

**Affiliations:** 1 University of Novi Sad, Faculty of Sciences, Department of Physics, 21000 Novi Sad, Serbia; dusica.krunic@df.uns.ac.rs; 2 University of Novi Sad, Faculty of Sciences, Department of Chemistry, Biochemistry and Environmental Protection, 21000 Novi Sad, Serbia; maria.savanovic@dh.uns.ac.rs (M.M.S.); sanja.armakovic@dh.uns.ac.rs (S.J.A.)

**Keywords:** poly(methyl methacrylate), sulfur-containing gases, GFN2-xTB, g-xTB, DFT, SAPT, MD, GFN-FF

## Abstract

The increasing emission of harmful gases into the atmosphere represents a major environmental challenge, driving the need for efficient air purification materials. Poly(methyl methacrylate) (PMMA) has emerged as a promising candidate due to its favorable physicochemical properties and adsorption potential. In this study, the interactions between PMMA and selected sulfur-containing pollutants (CH_3_SH, COS, CS_2_, H_2_S, and SO_2_) were systematically investigated using a multiscale computational approach. Initial structural exploration was performed using extended tight-binding (xTB) methods, followed by refinement at the density functional theory (DFT) level, while molecular dynamics (MD) simulations were employed to capture the dynamic behavior of the systems. The results suggest that all investigated gases exhibit attractive interactions with PMMA, with interaction strength strongly dependent on molecular polarity and electronic structure. Among the studied systems, SO_2_ shows the strongest binding, while CS_2_ exhibits the weakest interaction. Energy decomposition based on symmetry-adapted perturbation theory (SAPT) and electronic structure analyses suggest that electrostatic and donor–acceptor interactions play a dominant role for strongly interacting systems, whereas weaker interactions are primarily governed by dispersion forces.

## 1. Introduction

Due to significant technological and industrial progress, numerous negative effects on ecosystems and human health have emerged. Global warming, the greenhouse effect, acid rain, and the release of harmful gaseous chemicals into the atmosphere are considered leading environmental problems [1,2]. Human activities such as urbanization, increased consumption of fossil fuels, increased agricultural activities, and insufficient organization of waste disposal, contribute to these problems [3]. For this reason, from year to year, the preservation of environmental quality and air conditions has become an increasingly important research focus [4,5].

Harmful gases containing sulfur have been singled out as one of the most important air pollutants. Exposure to these gases directly affects the health of every person [6,7], but also leaves long-term negative consequences for the health of our planet [8,9]. Methanethiol (CH_3_SH), carbonyl sulfide (COS), carbon disulfide (CS_2_), hydrogen sulfide (H_2_S), and sulfur dioxide (SO_2_) are produced during the combustion of coal and oil, as well as in industrial plants, and due to volcanic eruptions. These gases contribute to the deterioration of air quality. They also contribute to soil pollution. They pose significant risks to human health as well as terrestrial and aquatic ecosystems [10,11]. It is extremely important that these harmful agents in the air are detected in time and removed to a greater extent, in order to preserve the environment, improve human health, and reduce climate change [12,13].

H_2_S and COS are among the most common impurities in natural gas and must be removed to meet strict emission standards [14]. H_2_S easily oxidizes, and in this way, SO_2_ is formed, while COS stimulates the formation of SO_2_ through hydrolysis. After photoexcitation, CS_2_ reacts with hydroxyl radicals to form SO_2_ [15,16,17]. SO_2_ is a major atmospheric pollutant associated with acid rain and smog, leading to severe environmental and health effects [18,19,20,21,22,23,24,25,26,27]. CH_3_SH, commonly emitted from wastewater treatment systems and landfills, is also problematic due to its toxicity and strong odor [28,29]. It is followed by an extremely unpleasant smell, which leads to an increasing need to develop systems for the purpose of destroying unpleasant smell pollution [30].

Polymethyl methacrylate (PMMA) has attracted increasing attention in environmental applications due to its stability, processability, and compatibility with various functional materials. It is widely used as an adsorbent, membrane component, and photocatalyst support, demonstrating potential in both water and air purification systems [31,32,33,34,35].

In recent years, PMMA has been widely explored as a promising material for gas separation and capture applications due to its favorable physicochemical properties, including good permeability, selectivity, and processability. For instance, PMMA-based mixed matrix membranes (MMMs) incorporating functionalized nanoparticles such as TiO_2_ have demonstrated substantially enhanced gas separation performance, particularly for CO_2_/N_2_ systems, highlighting the ability of PMMA to serve as an efficient separation medium when structurally modified [36]. Similarly, electrospun PMMA materials have shown remarkable CO_2_ capture capacity, fast sorption kinetics, and good recyclability, emphasizing their potential as solid sorbents under mild operating conditions [37]. Further, recent studies have explored PMMA-based composite membranes combined with advanced materials such as Matrimid and graphene oxide, achieving substantial improvements in permeability and selectivity for technologically relevant gas pairs, including CO_2_/N_2_ and CO_2_/CH_4_ [38].

Despite these advances, existing studies predominantly focus on macroscopic performance metrics, such as permeability, selectivity, and sorption capacity, often evaluated experimentally or through empirical optimization of composite systems. As a result, the fundamental atomistic mechanisms governing interactions between PMMA and small gas molecules remain insufficiently understood, particularly for environmentally relevant sulfur-containing pollutants. Further, most investigations are limited to a narrow set of gases, primarily CO_2_ and light hydrocarbons, while systematic comparative studies across chemically distinct sulfur species (e.g., CH_3_SH, COS, CS_2_, H_2_S, and SO_2_) are lacking. In addition, the dynamic behavior of these interactions under realistic conditions is rarely addressed, leaving a gap in understanding the interplay between static binding characteristics and time-dependent molecular motion.

From a materials design perspective, this limitation represents a significant challenge, as the absence of detailed atomistic insight restricts the rational development of polymer-based adsorbents and membranes. Understanding how molecular polarity, electronic structure, and geometry influence interaction strength is essential for tailoring materials with improved adsorption performance and selectivity.

Atomistic simulations represent a powerful approach for elucidating intermolecular interactions at a level of detail that is often inaccessible to experimental techniques alone. In recent years, the combination of different computational methods within a unified framework has emerged as a particularly effective strategy for achieving both accuracy and computational efficiency [39,40,41], although there is still no unique solution that can treat materials on larger scales with the same accuracy. In this context, density functional theory (DFT) has been widely employed for the investigation of adsorption phenomena and the rational design of materials [42,43], providing reliable insight into structural, energetic, electronic, and adsorption properties of molecular systems [44,45,46,47,48,49]. Nevertheless, capturing the full complexity of adsorption processes requires going beyond a single level of theory and incorporating complementary approaches that address different aspects of the interaction.

The relevance of atomistic calculations for PMMA systems is further supported by numerous computational studies. Early work by Subramanian et al. [50] employed molecular mechanics methods to investigate polypropylene and PMMA, successfully predicting their preferred conformations. In a more recent study, Shah et al. [51] used DFT at the B3LYP/6-31+G(d,p) level to examine structural, optical, and polymerization-related properties of PMMA. Atomistic calculations have also been used to rationalize classical experimental observations. For example, Paz et al. [52] applied DFT methods to interpret the pioneering findings of Michael Szwarc [53,54] regarding the influence of reactive site concentration on propagation rates and the so-called “self-killer” behavior of PMMA in living anionic polymerization. Their results indicated that the increase in propagation rate is associated with crosslinking effects, whereas the self-killer behavior is primarily governed by solvation phenomena rather than deactivation of reactive sites. In addition, Elbakyan and Zaporotskova [55] applied DFT methods to study the conductive properties of PMMA-based composites doped with carbon nanotubes, demonstrating that adsorption interactions between PMMA fragments and carbon nanotubes play a key role in the formation of stable polymer-nanotube complexes. These studies collectively demonstrate that atomistic calculations provide valuable insight into the structural, electronic, and mechanistic properties of PMMA. Furthermore, it is important to note that, in these studies, molecular models of PMMA used for quantum-mechanical calculations typically comprised between 1 and 15 monomer units, reflecting the necessary balance between computational cost and accuracy.

In this work, we adopt a multiscale computational strategy to investigate the interactions between the PMMA polymer and sulfur-containing gases (CH_3_SH, COS, CS_2_, H_2_S, and SO_2_), which represent a set of environmentally relevant pollutants. Initial structural exploration is performed using semiempirical extended tight-binding methods—GFN2-xTB and g-xTB. These methods enable efficient sampling of configurational space. The geometries are also optimized at the r^2^SCAN-3c level of theory, providing a balanced description of non-covalent interactions with improved accuracy. To obtain reliable energetic profiles, single-point energy calculations are carried out using the range-separated hybrid ωB97X-V functional. In addition, symmetry-adapted perturbation theory at the SAPT2 level is employed to decompose the interaction energies into physically meaningful components, allowing a detailed interpretation of the underlying interaction mechanisms. Finally, molecular dynamics simulations based on the GFN-FF force field are performed to capture the dynamic behavior of the PMMA-gas systems and to assess the stability of the interactions under realistic conditions. This interdisciplinary approach enables a detailed understanding of both the static and dynamic aspects of adsorption, providing deeper insight into the structure-interaction relationships governing the performance of PMMA as a potential material for air purification.

While our previous study focused on relatively simple and mostly symmetric molecules (H_2_, N_2_, O_2_, and H_2_O), the present work systematically investigates a chemically diverse set of sulfur-containing pollutants (CH_3_SH, COS, CS_2_, H_2_S, and SO_2_), which span a broader range of polarity, polarizability, and electronic structure. This enables a comparative analysis of interaction mechanisms across fundamentally different regimes, from weak dispersion-dominated interactions (e.g., CS_2_) to strongly polarized systems with significant electrostatic and donor–acceptor contributions (e.g., SO_2_).

Also, the present study is specifically focused on environmentally relevant sulfur-containing pollutants, which are of direct importance for air purification applications. By combining systematic chemical variation, detailed energy decomposition, and dynamic analysis, we believe this work provides new insight into structure-interaction relationships that are directly relevant for the rational design of polymer-based adsorbent materials.

## 2. Computational Details

In this work, geometrical optimization of all structures was firstly performed using the semiempirical GFN2-xTB and g-xTB methods, developed by Prof. Grimme et al. [56,57,58,59,60,61]. After that, the initial structures were optimized at the DFT level with the r^2^SCAN-3c composite method [62,63,64,65].

To account for different possible adsorption configurations, sulfur-containing gas molecules were positioned at multiple locations around the PMMA chain and in various orientations. Specifically, four distinct regions around the PMMA structure were considered, each combined with several possible orientations of the adsorbate molecules. This approach resulted in dozens of unique initial configurations, corresponding to molecular systems containing approximately 80–85 atoms.

For all calculations, a PMMA model consisting of five monomer units was employed. This model provides a suitable representation of the polymer environment while maintaining computational efficiency and sufficient flexibility to accommodate the investigated sulfur-containing gases. The chosen system size is consistent with previous studies, where PMMA models ranging from single monomer units to chains of up to 15 units have been successfully applied [51,55,66].

Following geometry optimization, binding energies were calculated using both semiempirical and DFT methods according to the following expression:

(1) $$E_{b}=E_{tot}\left(PMMA+SG\right)-E_{tot}\left(PMMA\right)-E_{tot}\left(SG\right)$$

where $E_{tot}\left(PMMA+SG\right)$ represents the total energy of the optimized system consisting of PMMA and the corresponding sulfur-containing gas (SG), $E_{tot}\left(PMMA\right)$ denotes the total energy of the optimized PMMA chain, while $E_{tot}(SG)$ represents the total energy of the optimized structure of the corresponding sulfur-containing gas.

For selected systems with the strongest binding, additional single-point calculations were performed at a higher level of theory to obtain accurate electron densities for the reduced density gradient (RDG) analysis. For this purpose, the ωB97X-V density functional [67] in combination with the def2-TZVP basis set [68,69] were used.

RDG analysis was used to generate both scatter plots and real-space RDG surfaces, which were further complemented by quantum theory of atoms in molecules (QTAIM) analysis, confirming the presence and nature of non-covalent interactions between PMMA and the investigated sulfur-containing gases.

All DFT calculations were performed using the ORCA 6.1.1 program package [70,71,72,73,74,75,76], developed by Neese and co-workers. RDG scatter plots and cube files required for RDG surface visualization were generated using the online RDG tool available within the atomistica.online 2025 molecular modeling platform [77,78], which utilizes the Multiwfn program version 3.8 [79,80,81,82] developed by Tian Lu. The Multiwfn software enables powerful analysis of electronic structure data obtained from various atomistic calculations.

All symmetry-adapted perturbation theory calculations of the second order (SAPT2) were performed in combination with jun-cc-pVDZ basis set, as implemented in the PSI4 code [83,84,85] for atomistic calculations.

The atomistica.online 2025 platform was also employed to perform GFN2-xTB calculations through an interface that utilizes the xtb code developed by Grimme and co-workers. All molecular dynamics (MD) simulations were performed using the GFN-FF force field [86]. The simulations were performed at a temperature of 300 K with a time step of 2 fs, and a total production time of 10 ns. All MD simulations were performed within the canonical (NVT) ensemble, with the xtb accuracy parameter set to 2.0. The initial configurations for the MD simulations were obtained from geometries previously optimized at the GFN2-xTB level of theory, ensuring that the systems were initialized close to their local energy minima. Due to this, no separate equilibration phase was introduced, and the simulations were directly propagated. MD simulations were carried out using the xtb code and its GUI from atomistica.online. Atomistica and its online and desktop tools are freely available at https://atomistica.online. Visualization of frontier molecular orbitals and calculation of radial distribution functions were performed using the VMD program [87,88,89,90,91,92]. All molecular structures, including initial geometries and those obtained after geometry optimizations at the GFN2-xTB, g-xTB, and r^2^SCAN-3c levels of theory, are freely available for download from the GeoHub service of Atomistica (https://geohub.atomistica.online accessed on 4 May 2026).

## 3. Results and Discussion

### 3.1. Structural Considerations

The discussion begins with the structural models of the investigated molecular systems. To explore different molecular geometries of the PMMA gas systems (CH_3_SH, COS, CS_2_, H_2_S or SO_2_), each gas molecule was placed around the central part of the polymer chain at four different locations. At each of the mentioned locations, the gases are placed in two (SO_2_, H_2_S, CS_2_) or three (COS, CH_3_SH) different orientations: horizontally and vertically in relation to the polymer chain. This resulted in a total of forty-eight different configurations for all five gases.

**Figure 1:**
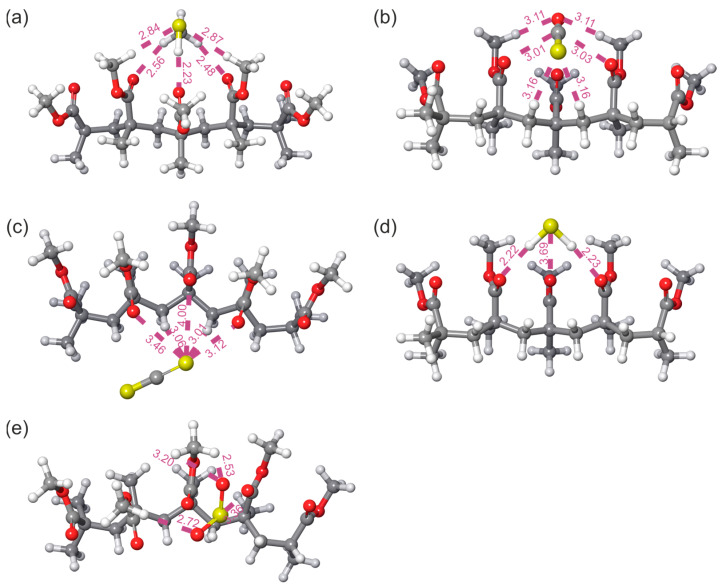
r^2^SCAN-3c optimized systems of (**a**) PMMA-CH_3_SH, (**b**) PMMA-COS, (**c**) PMMA-CS_2_, (**d**) PMMA-H_2_S, (**e**) PMMA-SO_2_ with indicated distances (Å) between PMMA and sulfur-containing pollutants.

The geometrically optimized systems obtained by the r^2^SCAN-3c method with the strongest binding energies are shown in Figure 1. The pink dashed lines indicate the specific distances that represent the closest distances between PMMA and gas molecules.

The shortest intermolecular distance is observed in the PMMA-H_2_S system (Figure 1d), between the hydrogen atom of H_2_S and the oxygen atom of the PMMA chain, with a value of 2.22 Å. According to the obtained results, the distance between PMMA and the considered molecules was in the range between 4.00 Å and 2.22 Å. The largest distances are observed for the PMMA-CS_2_ system, consistent with its lowest binding energy among the investigated systems.

### 3.2. Binding Energies

When evaluating the potential of a material to act as an adsorbent, separator, or membrane for the removal of harmful gases, the binding energy represents one of the key descriptors of adsorption performance. It provides a quantitative measure of the interaction strength between the adsorbent and the target molecule and, consequently, insight into the feasibility of both adsorption and regeneration processes. In the context of air purification, an optimal balance is required: interactions must be sufficiently strong to ensure effective capture, yet not so strong as to prevent desorption and material reuse.

To elucidate the adsorption behavior of sulfur-containing gases on the PMMA surface, binding energies were calculated using both semiempirical (GFN2-xTB) and density functional theory (r^2^SCAN-3c) approaches. The use of multiple levels of theory enables a more detailed and reliable description of the interaction, allowing us to assess both methodological consistency and the robustness of observed trends.

While quantitative differences of several kcal/mol are observed between the two methods, especially for strongly interacting systems such as SO_2_, these deviations fall within the expected accuracy range of the employed computational approaches. Further, both methods yield consistent qualitative trends, supporting the reliability of the predicted adsorption behavior. The calculated binding energies are summarized in Table 1.

The binding energies obtained using GFN2-xTB, the just recently developed g-xTB method, and the r^2^SCAN-3c method show overall good qualitative agreement. All three approaches consistently predict attractive interactions for the investigated sulfur-containing pollutants and identify SO_2_ as the strongest interacting species and CS_2_ as the weakest. The calculated binding energies for the PMMA-H_2_S, PMMA-COS, and PMMA-CH_3_SH systems fall within a comparable energy range (approximately −6.02 to −11.34 kcal/mol, depending on the method). At the r^2^SCAN-3c level, the PMMA-H_2_S and PMMA-CH_3_SH systems exhibit very similar binding energies (−8.16 kcal/mol and −8.12 kcal/mol, respectively), indicating comparable interaction strengths.

A deviation in the relative ordering is observed when comparing GFN2-xTB with r^2^SCAN-3c results, particularly for the PMMA-CH_3_SH and PMMA-COS systems, where GFN2-xTB predicts a reversed trend. This behavior can be attributed to the nature of the interactions governing these systems. In contrast to strongly interacting species such as SO_2_, where electrostatic and donor-acceptor interactions play a dominant role, the interactions involving CH_3_SH and CS_2_ are weaker and largely governed by dispersion forces. In such cases, the calculated binding energies are highly sensitive to subtle differences in molecular orientation and intermolecular distances. Due to the conformational flexibility of the PMMA chain, different computational methods may favor slightly different local minima on the potential energy surface, leading to variations in interaction geometries and corresponding binding energies.

Importantly, the inclusion of g-xTB results provides additional insight into this discrepancy. The g-xTB method reproduces the same ordering of interaction strengths as r^2^SCAN-3c (PMMA-SO_2_ > PMMA-H_2_S > PMMA-CH_3_SH > PMMA-COS > PMMA-CS_2_), including the correct relative positioning of the PMMA-CH_3_SH and PMMA-COS systems. This indicates that the deviation observed for GFN2-xTB arises from limitations in its treatment of weak, dispersion-dominated interactions rather than from intrinsic ambiguity in the underlying chemistry.

Furthermore, the r^2^SCAN-3c method incorporates an advanced treatment of dispersion interactions through D4 corrections, as well as geometric counterpoise (gCP) corrections for basis set superposition error, which contributes to a more reliable description of noncovalent interactions. The improved agreement between g-xTB and r^2^SCAN-3c suggests that g-xTB provides a more balanced treatment of electrostatic and dispersion contributions compared to GFN2-xTB. Consequently, the combined use of these methods strengthens the overall consistency and reliability of the obtained trends in binding energies across the investigated systems.

The obtained binding energies provide important insight into the practical applicability of PMMA as a material for gas capture and separation. The predicted interaction strengths fall within a moderate energy range, which is particularly favorable for adsorption-based processes, as it ensures a balance between efficient gas capture and feasible desorption. This suggests that PMMA might have the potential to act as a reusable adsorbent, capable of binding sulfur-containing pollutants without permanent retention. The observed trend in interaction strength further shows that adsorption may be strongly influenced by the polarity and electronic structure of the gas molecules, with more polar species such as SO_2_ exhibiting substantially stronger interactions due to enhanced electrostatic contributions. In contrast, weaker binding observed for less polar systems, such as CS_2_, points to a larger role of dispersion-driven interactions.

This trend suggests that adsorption on PMMA is governed by a balance between electrostatic and dispersion interactions, which can be tuned through structural modification of the polymer.

### 3.3. Noncovalent Interactions

**Figure 2:**
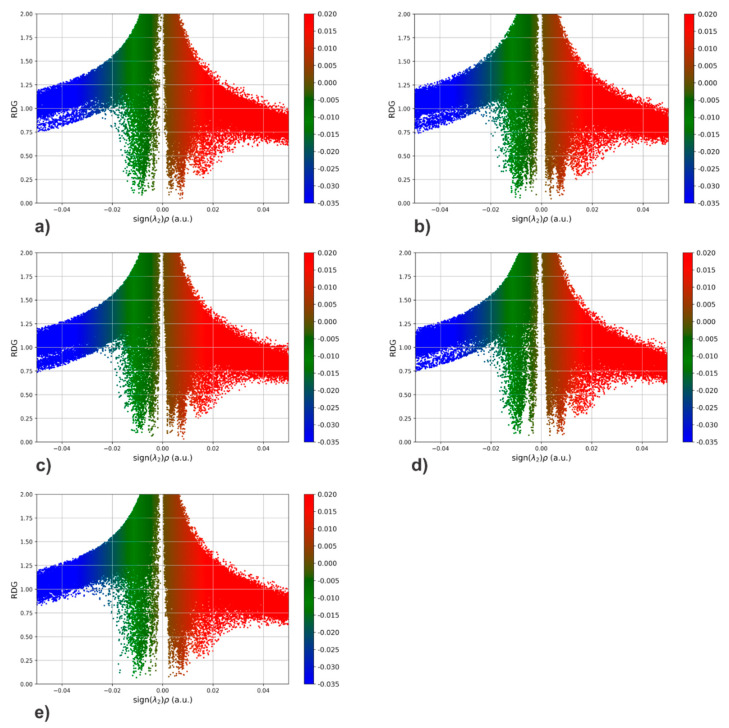
RDG scatter plots of (**a**) PMMA-CH_3_SH, (**b**) PMMA-COS, (**c**) PMMA-CS_2_, (**d**) PMMA-H_2_S, and (**e**) PMMA-SO_2_.

To gain a deeper insight into the binding between PMMA and the considered harmful gases, we performed a detailed examination of the non-covalent interactions that occur between the mentioned molecules. The identification of non-covalent interactions was made thanks to the analysis of the electron density between all atoms [93]. We applied the RDG approach, which gave us the opportunity to examine the interactions mentioned in detail. Based on the analysis of the RDG value in relation to the sign of (λ_2_)*ρ*, we can identify the type of interaction, such as hydrogen bonding, π-π stacking, or weak van der Waals interaction. A detailed view of non-covalent interactions obtained based on the analysis of RDG values versus the sign of (λ_2_)*ρ* values between the studied systems in this study is shown in Figure 2.

RDG scatterplots are color-mapped according to the strength of non-covalent interactions. The blue dots symbolize a strong interaction, that is, the formation of a hydrogen bond (λ_2_ < 0, ρ > 0). Red dots represent strong repulsion (λ_2_ > 0, ρ > 0), while weak van der Waals interactions are shown by green dots (λ_2_ ≈ 0, ρ ≈ 0).

In the case of the PMMA-SO_2_ system (Figure 2e), a relatively higher population of blue regions is observed compared to the other systems, suggesting the presence of stronger attractive interactions. In contrast, the PMMA-CS_2_ system (Figure 2c) shows fewer such regions, consistent with weaker intermolecular interactions.

It should be noted that RDG analysis provides qualitative insight into the presence and spatial distribution of noncovalent interactions, rather than a direct quantitative measure of interaction strength. Nevertheless, the observed trends are in agreement with the calculated binding energies.

### 3.4. Molecular Dynamics Simulations

MD simulations, in addition to binding energies and analysis of non-covalent interactions, represent another important computational technique. With their help, it is possible to obtain a thorough representation of the interactions between PMMA and selected sulfur-containing gases. These simulations provide additional insight into dynamic properties, that is, how gas molecules behave in the presence of polymer chains. Using MD simulations, it is possible to effectively model the complex behavior of particles at the atomic level and analyze their adsorption, diffusion, and solubility within the polymer itself. In order to examine the potential of PMMA as a possible adsorber of harmful gases containing sulfur, understanding the mentioned interactions is of great importance.

In this paper, MD simulations were performed using the GFN-FF force field on systems consisting of one PMMA chain surrounded by ten molecules of the considered gases. The choice of an appropriate force field is crucial for accurately describing atomic interactions and system dynamics. The GFN-FF force field is characterized by speed, precision, efficiency, and flexibility.

It should be noted that the simulated system, consisting of a single PMMA chain and a limited number of gas molecules, represents a simplified model of a polymer–gas environment. The primary aim of these simulations is to provide insight into local interaction patterns and dynamical behavior at the atomistic level, rather than to reproduce macroscopic adsorption properties.

**Figure 3:**
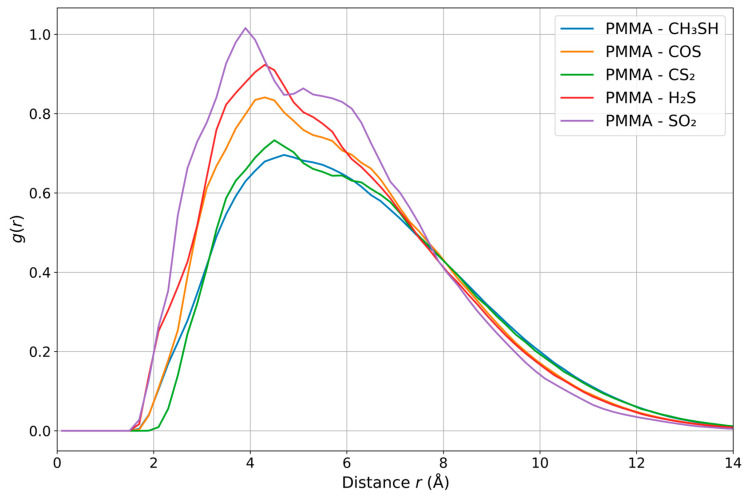
RDFs of PMMA against CH_3_SH, COS, CS_2_, H_2_S and SO_2_ molecules.

The radial distribution function (RDF) provides us with quantitative information about the spatial distribution of atoms or molecules around a reference point. It represents the probability of finding one particle relative to the distance from another. RDF diagrams provide us with significant insight into the arrangement of gas molecules around the PMMA chain, highlighting the affinity and effects of a given gas located near the polymer. Figure 3 shows the RDF with respect to the distance between the PMMA and the considered gas molecules.

The radial distribution functions (RDFs) presented in Figure 3 reveal distinct differences in the interaction behavior of sulfur-containing gases with the PMMA chain. The PMMA-SO_2_ system exhibits the most pronounced and well-defined peak, corresponding to the highest value of g(r) (1.01), indicating a strong and highly structured interaction. The position of this peak at approximately 3.88 Å suggests a preferred interaction distance between SO_2_ and the PMMA surface, consistent with its strong binding affinity observed in the DFT analysis.

Moderately intense peaks are observed for the PMMA-H_2_S and PMMA-COS systems, indicating intermediate interaction strength. Although both systems display similar peak intensities, differences in peak positions reflect variations in their preferred interaction distances. These results suggest that while both gases interact favorably with PMMA, their spatial organization and interaction modes differ. In contrast, the PMMA-CS_2_ and PMMA-CH_3_SH systems exhibit less pronounced RDF peaks and broader distributions, indicating weaker and less structured interactions with the polymer. The larger average distances and lower peak intensities suggest reduced affinity toward the PMMA surface, which is consistent with their lower binding energies. The obtained results are consistent with previous results on binding energies based on DFT calculations.

### 3.5. Frontier Molecular Orbitals

**Figure 4:**
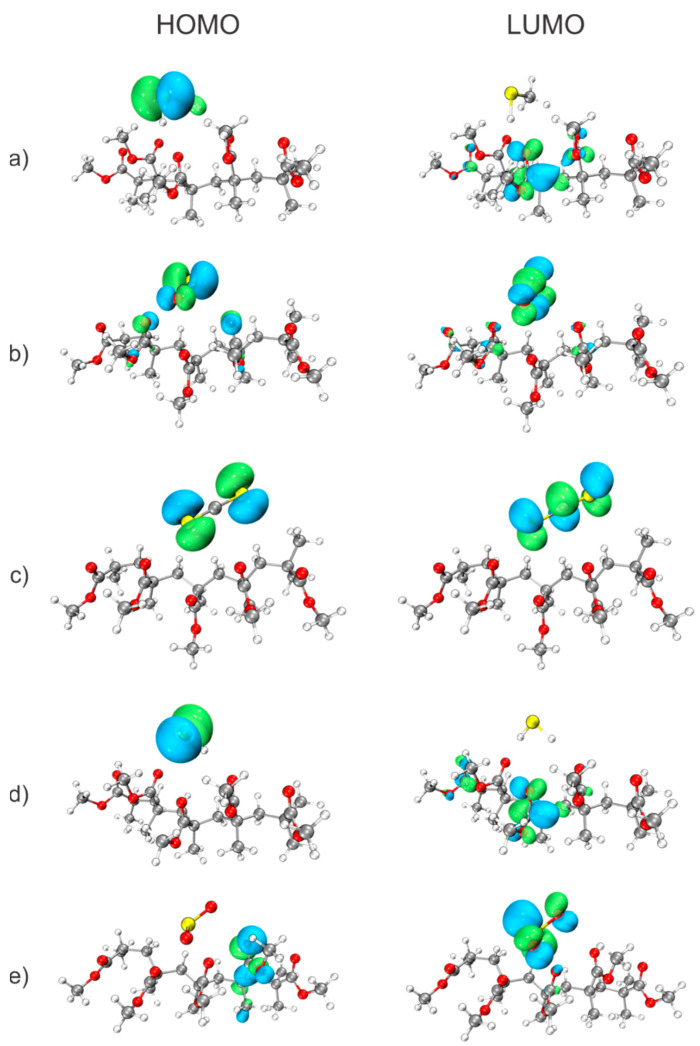
HOMOs and LUMOs for adsorption of molecules—(**a**) CH_3_SH, (**b**) COS, (**c**) CS_2_, (**d**) H_2_S, (**e**) SO_2_ by PMMA.

Using DFT calculations, the frontier molecular orbitals, highest occupied molecular orbital (HOMO) and lowest unoccupied molecular orbital (LUMO), were analyzed to gain insight into the electronic characteristics of the studied systems. The HOMO corresponds to the highest-energy orbital containing electrons and is associated with the ability of a molecule to act as an electron donor. Conversely, the LUMO represents the lowest-energy vacant orbital and is related to the electron-accepting capability of the molecule. HOMO and LUMO molecular orbitals are the most important molecular orbitals that contribute to the global reactivity properties of organic molecules [94,95]. In this paper, we have visualized the distribution of HOMO and LUMO molecular orbitals, which can be seen in Figure 4. This provides insight into studying the charge transfer between the adsorber and the adsorbate.

The results presented in Figure 4 reveal clear trends in the distribution of frontier molecular orbitals. In most cases, the HOMO is predominantly localized on the adsorbate molecules, which may suggest that the sulfur-containing gases tend to act as electron donors in their interaction with PMMA. A notable exception is observed for the SO_2_ system, which also exhibits the strongest binding energy. In this case, the HOMO is localized on the PMMA fragment, while the LUMO is entirely concentrated on the SO_2_ molecule. This spatial separation of frontier orbitals may indicate a possible donor-acceptor interaction pattern, where PMMA could acts as an electron donor and SO_2_ as an electron acceptor, which is consistent with the strong interaction energy obtained for this system.

In contrast, for the weakest interacting system, PMMA-CS_2_, both the HOMO and LUMO are fully localized on the CS_2_ molecule. This suggests limited orbital interaction between the interacting species and indicates that any charge-transfer contributions, if present, are likely small. Consequently, the interaction in this case is expected to be dominated by weaker non-covalent contributions, such as dispersion and electrostatics, in agreement with the low binding energy.

For the remaining systems, the frontier orbitals are distributed over both PMMA and the adsorbate, suggesting some degree of orbital interaction and possible donor–acceptor character. This is also evident for the PMMA-CH_3_SH system, which exhibits the strong binding energy according to r^2^SCAN-3c calculations. In this case, the HOMO is localized on CH_3_SH, while the LUMO is primarily associated with the PMMA fragment, which may be consistent with electron density transfer from the adsorbate toward the polymer. A similar qualitative interpretation can be made in the case of interaction between PMMA and H_2_S, where the spatial distribution of frontier orbitals suggests a comparable interaction pattern, also in agreement with the calculations of binding energies.

It should be emphasized that the visualization of frontier molecular orbitals provides qualitative insight only, and does not constitute direct evidence of charge transfer. Therefore, the observed distributions should be interpreted as indicative of possible interaction patterns rather than definitive proof of electronic charge redistribution.

### 3.6. SAPT Analysis

To gain a deeper insight into the nature of the noncovalent interactions between the investigated systems, the interaction energy was further analyzed using symmetry-adapted perturbation theory (SAPT). This approach enables decomposition of the total interaction energy into physically meaningful components, namely electrostatic (EL), exchange (EX), induction (I), and dispersion (D) contributions. In the present study, the SAPT2 level of theory was applied, which demanded from us to reduce the size of molecular models by truncating the terminal segments of the polymer chain, while preserving the local interaction region between PMMA and the molecule intact. The EL term arises from Coulombic interactions between the charge distributions of the interacting monomers, while the EX component reflects the Pauli repulsion originating from wavefunction overlap and the antisymmetry requirement of the dimer wavefunction. The induction term accounts for polarization and charge-transfer effects, and the dispersion contribution represents electron-correlation-driven interactions. While EL, I, and D generally contribute attractively to the general stabilization, the EX component is inherently repulsive. Decomposition of the interaction energy into these components provides a clearer understanding of the dominant forces governing the stability and structure of the analyzed complexes.

**Figure 5:**
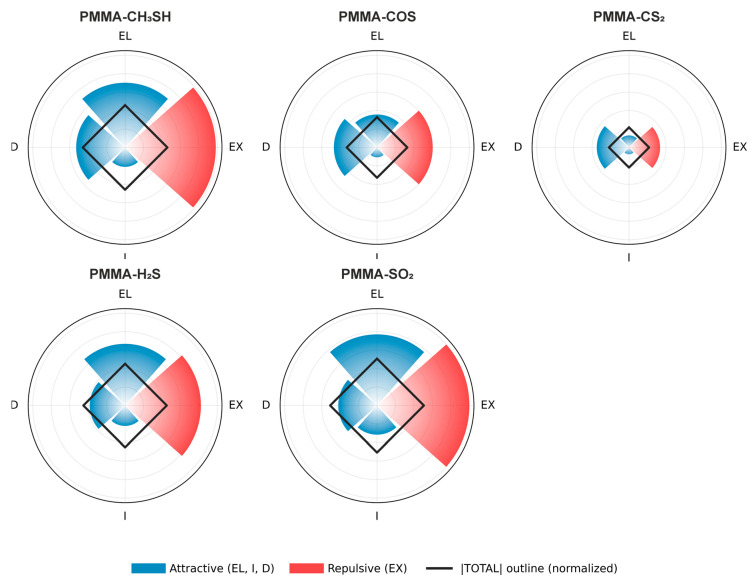
Radar plot of SAPT2 interaction energy components between sulfur-containing pollutant and PMMA.

To perform a compact and intuitive comparison of SAPT2 energy decomposition across different PMMA-pollutant systems, the interaction components were visualized using customized radar plots (“energy fingerprints”), as presented in Figure 5, while numerical values used to generate these plots are provided in the Supplementary Materials.

Each sector of the radar corresponds to one SAPT2 contribution (EL, EX, I, and D), while the radial length reflects the normalized absolute magnitude of the respective term, allowing direct comparison between systems on a common scale. Attractive components (EL, I, D) and the repulsive exchange term (EX) are distinguished by contrasting colors to preserve their physical meaning. In addition, the magnitude of the total interaction energy is indicated by an overlaid circular outline. All numerical values regarding SAPT2 calculations are provided in the Table S1 of the Supplementary Materials.

Results provided in Figure 5 show that the dominant attractive contribution is the EL term, particularly for more polar molecules. This is most pronounced for PMMA-SO_2_ (−10.37 kcal/mol), followed by PMMA-CH_3_SH (−9.44 kcal/mol) and PMMA-H_2_S (−8.99 kcal/mol), confirming that permanent charge distributions and dipole–dipole interactions play a central role in stabilizing these intermolecular systems. In contrast, PMMA-CS_2_ exhibits the weakest electrostatic contribution (−1.76 kcal/mol), consistent with its non-polarity.

The D component is also a major stabilizing factor across all systems, with values ranging from −4.70 to −7.13 kcal/mol. For less polar systems such as CS_2_ and COS, dispersion becomes relatively more important, in some cases comparable to or exceeding the electrostatic contribution. The I term remains smaller but non-negligible (−0.99 to −4.25 kcal/mol), with the strongest contribution observed for SO_2_, reflecting its high polarizability and strong response to the PMMA electrostatic field.

The EX term represents the dominant repulsive contribution, with values between +4.52 and +13.45 kcal/mol. Larger exchange contributions are observed for systems exhibiting stronger attractive interactions, such as PMMA-SO_2_ and PMMA-CH_3_SH, highlighting the balance between stabilizing (EL + D + I) and destabilizing (EX) terms that determines the final interaction energy.

When considering the total interaction energies, PMMA-SO_2_ (−6.83 kcal/mol) is identified as the most strongly bound system, followed by PMMA-CH_3_SH (−6.17 kcal/mol) and PMMA-H_2_S (−6.08 kcal/mol), which exhibit very similar interaction strengths. PMMA-COS (−4.42 kcal/mol) and PMMA-CS_2_ (−2.93 kcal/mol) show weaker interactions overall, consistent with their reduced polarity and smaller electrostatic contributions.

Overall, these results confirm that electrostatic interactions dominate in more polar systems, while dispersion contributions become increasingly important for less polar molecules, providing a clear and physically grounded description of polymer–gas interactions.

A comparison between the interaction energies obtained at the r^2^SCAN-3c and SAPT2 levels shows an overall consistent qualitative agreement across all systems. Both methods predict PMMA-SO_2_ as the most strongly bound intermolecular system, while PMMA-COS and PMMA-CS_2_ exhibit weaker interactions. For PMMA-CH_3_SH and PMMA-H_2_S, both approaches yield very similar interaction energies, although a slight difference in ordering is observed between the two methods. Given that the energy differences between these two systems are extremely small (on the order of ~0.05–0.1 kcal/mol), this variation is not considered significant and reflects the sensitivity of closely competing interactions to the level of theory.

The SAPT2 interaction energies are systematically less negative than the corresponding DFT binding energies, which is expected due to methodological differences in the treatment of intermolecular interactions. However, the differences between the two methods remain relatively small and uniform (approximately 2–4 kcal/mol), confirming a high degree of consistency in the predicted interaction strengths. Overall, both approaches provide a coherent and mutually supportive description of the PMMA-gas interactions.

## 4. Conclusions

This study investigates the interactions between PMMA and selected sulfur-containing gases (CH_3_SH, COS, CS_2_, H_2_S, SO_2_) with the aim of providing atomistic-level insight into their interaction behavior. The computational study of this work included semiempirical, DFT and SAPT2 calculations, together with MD simulations.

The results consistently demonstrate that all investigated gases interact attractively with the PMMA polymer, with interaction strength strongly dependent on the polarity and electronic structure of the adsorbate. Among the studied systems, SO_2_ exhibits the strongest binding, which is supported across all applied computational approaches, including binding energy analysis, RDG and QTAIM characterization, molecular orbital distribution, MD simulations, and SAPT2 energy decomposition. In contrast, CS_2_ shows the weakest interaction, characterized by reduced orbital overlap and a dominant contribution of dispersion forces.

Energy decomposition analysis reveals that electrostatic interactions play a dominant role in stabilizing strongly interacting systems, while dispersion contributions become increasingly important for less polar molecules. The interplay between attractive (electrostatic, induction, and dispersion) and repulsive (exchange) components governs the overall stability of the complexes, providing a detailed mechanistic understanding of PMMA-gas interactions.

These findings suggest that the balance between electrostatic and dispersion contributions plays an important role in governing polymer-gas interactions.

Importantly, the calculated binding energies fall within a moderate range, indicating reversible interaction behavior at the molecular level. The consistency between DFT, SAPT2, and MD results further supports the reliability of the multiscale approach.

Finally, this study provides a detailed atomistic-level understanding of the interaction mechanisms governing PMMA-gas systems and highlights general interaction trends within the studied model system. The insights obtained here may serve as a basis for future studies involving more complex and realistic systems.

## Figures and Tables

**Table 1:** Binding energies (kcal/mol) between PMMA and sulfur-containing pollutants using two methods.

System	GFN2-xTB	g-xTB	r^2^SCAN-3c
PMMA-CH_3_SH	−6.66	−8.45	−8.12
PMMA-COS	−9.58	−6.41	−6.02
PMMA-CS_2_	−2.27	−4.06	−5.44
PMMA-H_2_S	−11.34	−9.68	−8.16
PMMA-SO_2_	−20.64	−12.76	−11.16

## Data Availability

The original contributions presented in the study are included in the article/Supplementary Materials. Further inquiries can be directed to the corresponding author.

## Supplementary Materials

The following supporting information can be downloaded at: https://www.mdpi.com/article/10.3390/polym18101199/s1, Table S1: Numerical values of the interaction energy components as obtained by the SAPT2 calculations with jun-cc-pVDZ basis set. All values are given in kcal/mol.
