# Supplementary material for: Atomistic Insights on Interactions Between Sulfur-Containing Pollutants and PMMA: A Semiempirical, DFT, SAPT and Molecular Dynamics Study
Source: Polymers (Basel). 2026 May 14;18(10):1199. doi: 10.3390/polym18101199 (PMC13210670; doi:10.3390/polym18101199)
Supplement: Supplementary file 1 [file polymers-18-01199-s001.zip › polymers-4277892-supplementary.pdf]

Atomistic Insights on Interactions Between Sulfur-Containing Pollutants and PMMA: A Semiempirical, DFT, SAPT and Molecular Dynamics Study

Dušica Krunić[1], Stevan Armaković[1,*], Maria M. Savanović[2], Sanja J. Armaković[2]

[1]University of Novi Sad, Faculty of Sciences, Department of Physics, Novi Sad, Serbia; dusica.krunic@df.uns.ac.rs

[2]University of Novi Sad, Faculty of Sciences, Department of Chemistry, Biochemistry and Environmental Protection, Novi Sad, Serbia; maria.savanovic@dh.uns.ac.rs, sanja.armakovic@dh.uns.ac.rs

* Correspondence: stevan.armakovic@df.uns.ac.rs

SUPPLEMENTARY MATERIALS

1. Optimized geometries

All molecular structures, including initial geometries, structures obtained after geometry optimizations at the GFN2-xTB, g-xTB, and r^{2}SCAN-3c levels of theory, as well as those selected for subsequent single-point energy calculations, are freely available for download from the GeoHub service of Atomistica (https://geohub.atomistica.online).

2. SAPT2 results

Table S1. Numerical values of the interaction energy components as obtained by the SAPT2 calculations with jun-cc-pVDZ basis set. All values are given in kcal/mol

System	EL	EX	I	D	TOTAL
PMMA-CH3SH	-9.44	13.22	-2.83	-7.13	-6.17
PMMA-COS	-4.81	8.11	-1.44	-6.29	-4.42
PMMA-CS2	-1.76	4.52	-0.99	-4.70	-2.93
PMMA-H2S	-8.99	11.05	-2.98	-5.15	-6.08
PMMA-SO2	-10.37	13.45	-4.25	-5.65	-6.83
